# Exploring the Role of Activating Agents on the Electrocatalytic Activity of Grape Pomace‐Derived Catalysts for the Oxygen Reduction Reaction

**DOI:** 10.1002/smsc.70300

**Published:** 2026-05-13

**Authors:** Gloria Flores‐Gómez, Gabriel Abarca, Cristian Silva, Gustavo Chacón‐Rosales, José H. Zagal, Srinu Akula, Kaido Tammeveski, F. Javier Recio, Ricardo Venegas, Karina Muñoz‐Becerra

**Affiliations:** ^1^ Centro Integrativo de Biología y Química Aplicada (CIBQA) Facultad de Ciencias de la Salud Universidad Bernardo O’Higgins Santiago Chile; ^2^ Instituto de Tecnología Química (ITQ) Consejo Superior de Investigaciones Científicas‐Universitat Politècnica de València Valencia Spain; ^3^ Departamento de Química de los Materiales Facultad de Química y Biología Universidad de Santiago de Chile Santiago Estación Central Chile; ^4^ Institute of Chemistry University of Tartu Tartu Estonia; ^5^ Departamento de Química Física Aplicada Facultad de Ciencias Universidad Autónoma de Madrid Madrid España; ^6^ Escuela de Tecnología Médica Facultad de Ciencias de la Salud Universidad Bernardo O’Higgins Santiago Chile; ^7^ Escuela de Kinesiología, Facultad de Ciencias de la Salud Universidad Bernardo O'Higgins Santiago Chile

**Keywords:** biomass valorization, electrocatalysis, Fe‐N‐C catalysts, nonprecious metal catalysts, oxygen reduction reaction, wine grape pomace

## Abstract

The development of efficient and sustainable nonprecious metal catalysts for the oxygen reduction reaction (ORR) is central to advancing next‐generation fuel cells and metal‐air batteries. Fe‐N‐C catalysts derived from biomass have emerged as promising candidates, yet the influence of activation chemistry on the active‐site formation and electrocatalytic performance remains insufficiently explored. Here, we unveil how KOH (strong base) and ZnCl_2_ (Lewis acid) modulate the chemical activation of wine grape pomace, a readily available agri‐food residue, to generate Fe‐N‐C electrocatalysts through a one‐step synthesis without external nitrogen precursors. KOH activation promotes extensive micro‐ and mesoporosity and favors the in situ development of well‐coordinated Fe‐N_x_ moieties, yielding a large electrochemically active surface area and a high ORR onset potential of 0.87 V versus reversible hydrogen electrode, comparable to leading biomass‐derived Fe‐N‐C catalysts. In contrast, ZnCl_2_ activation reduced the density of active sites, resulting in diminished ORR activity. This work provides insight into how activation pathways govern porosity evolution, Fe‐N_x_ site formation, and ORR performance. More broadly, it demonstrates the effective valorization of winemaking residues into functional electrocatalysts, offering a scalable and sustainable route for materials design in electrochemical energy conversion technologies.

## Graphical abstract

This work explores the influence of KOH (alkaline agent) and ZnCl_2_ (pore‐generating agent) on the one‐step chemical activation of wine grape pomace to produce Fe‐N‐C catalysts for the oxygen reduction reaction (ORR). The analyses reveal that KOH activation generates a highly porous structure with Fe‐N_x_ sites, leading to a larger electrochemically active surface area and superior ORR electrocatalytic activity.

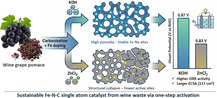

## 1 Introduction

Energy conversion technologies play a crucial role in energy transitions [1, 2]. However, the oxygen reduction reaction (ORR) remains one of the major kinetic bottlenecks in fuel cells and metal‐air batteries [3, 4, 5, 6, 7, 8]. demanding highly active, durable, and economically viable electrocatalysts. While Pt group metal‐based catalyst materials have long been considered the benchmark for ORR, their high cost, scarcity, and sensitivity to fuel impurities continue to hinder the large‐scale deployment of electrochemical energy devices [9, 10, 11]. Several alternatives have emerged in the literature focusing on nonprecious metal catalysts, metal‐free catalysts, and biomass‐derived materials as more sustainable and cost‐effective options for the large‐scale applications [12, 13, 14, 15, 16, 17, 18, 19, 20].

Pyrolyzed transition metal‐nitrogen‐carbon (M‐N‐C) catalysts stand out for their high electrocatalytic activity for ORR, as well as their long‐term stability [21, 22]. M‐N‐C materials commonly comprise M‐N_x_ centers, N‐C defects, and metal‐based nanoparticles integrated into graphitized carbon matrices, presenting distinctive catalytic selectivity [12, 13, 23, 24]. Their synthesis typically involves pyrolysis under an inert atmosphere using carbon, nitrogen, and metal precursors, or alternatively, the use of templating agents such as metal‐organic frameworks, which can also serve as sources of M, N, and C [25, 26]. Biomass‐derived carbons have gained significant attention as sustainable precursors for M‐N‐C catalysts, offering intrinsic heteroatoms, hierarchical porosity, and low environmental footprint [15, 27, 28, 29, 30]. This approach not only valorizes biowaste but also contributes to the transition to more sustainable, cost‐effective, and eco‐friendly energy systems, addressing both environmental and economic challenges [31, 32]. However, the mechanisms by which different activation chemistries govern porosity development, nitrogen functionalities, and the formation of catalytically relevant Fe‐N_x_ moieties remain insufficiently understood. This gap limits the rational design of next‐generation catalysts with maximized site density and optimized mass transport. Biowaste, or biomass waste, refers to residual materials from agricultural and forestry industries. Composed primarily of carbohydrates and polysaccharides, biowaste consists mainly of C, H, O, and N elements, making it a promising and sustainable precursor for C–N and M–N–C electrocatalysts [15, 28, 29]. Pyrolysis is a widely employed method that enables the thermal decomposition of biomass, gradually removing volatile fragments and generating structural defects through aromatic condensation [33, 34, 35]. Pyrolysis parameters (time, temperature, and heating rate) critically influence the electrocatalytic performance of biowaste‐derived materials. Activation also plays a role in tuning the final chemical composition of the material [35, 36]. Chemical activation is generally preferred over physical activation due to its lower required pyrolysis temperatures [35, 36, 37]. Physical activation usually involves pyrolysis between 700 and 1200°C under oxidizing atmospheres (steam, CO_2_, air). In contrast, chemical activation involves impregnating the biomass with a chemical agent such as ZnCl_2_, H_3_PO_4_, NaOH, KOH, or K_2_CO_3_, prior to pyrolysis, which is carried out at temperatures between 400 and 900°C [37, 38, 39, 40].

Pore formation during chemical activation is attributed to the oxidative removal of weakly bonded carbon atoms and volatilization of reducing gases (H_2_, CO, CO_2_). However, the activation mechanism depends on the specific reagent used. For example, KOH, as a strong base, chemically reacts with carbon atoms containing unpaired electrons and with acidic terminal groups in the biomass [38, 41]. Its activation mechanism begins with KOH dehydration at temperatures near 350°C, forming molten phases such as K_2_O and K_2_O_2_, along with free K, which intercalates into the carbonaceous matrix [39]. Then, between 450 and 650°C, residual KOH further reacts with defect‐rich carbon sites, releasing free K and reductive gases (H_2_ and CO), which promotes the formation of micropores and macropores within the carbon network. At higher temperatures (above 650°C), the remaining K‐containing phases (KOH, K_2_O_2_, free K) react with CO and CO_2_ gases generated during the carbonaceous polymerization, primarily forming K_2_CO_3_, which is subsequently removed from the graphitized product through acid leaching [37, 41].

Alternatively, ZnCl_2_, which behaves as a Lewis acid, acts as a dehydrating agent, promoting carbon biomass polymerization [42, 43]. At temperatures between 170 and 450°C, ZnCl_2_ interacts with water molecules released during the deep drying process, forming a hydrated ZnOCl_2_·2H_2_O salt. Above 450 °C, this salt is dehydrated, forming molten ZnO, which, at temperatures exceeding 650°C, reacts with the charcoal network. This reaction induces aromatic carbon polymerization and releases free Zn along with CO and CO_2_ gases, contributing to the porosity of the final graphitized material [44, 45, 46]. The remaining Zn‐phases at the final stage of the thermal treatment are removed by the acid washing of the obtained graphitized material.

Numerous studies have investigated the selective activation of different types of biomass waste to develop low‐cost catalysts for ORR [15, 28, 29, 33, 36, 47, 48, 49, 50]. Introducing N‐rich agents into biowaste further enhances its catalytic performance for energy conversion reactions [51]. N‐sites promote the formation of highly active M‐N_*x*_ sites when transition metal salts are used as metal doping agents (M–N–C catalysts) [37, 38, 39, 40]. Although higher N content is generally associated with enhanced activity in self‐doped biomass‐derived materials [52]. Dessalle et al*.* [28] encountered an inverse trend, observing a negative correlation between N content and specific surface area. However, when Fe is introduced during the activation process, the ORR activity is linked to the synergistic effect between N‐functionalities and Fe‐N_x_ sites, whose availability increases with porosity [28, 51, 53]. Additionally, metallic nanoparticles formed during thermal transformation (Fe_3_C, Fe_3_O_4_, among others) can directly influence the electrocatalytic activity of these materials [28].

Wine grape pomace (WP), a widely available agro‐industrial residue [54, 55], has emerged as a highly attractive feedstock due to its nitrogen‐rich polyphenolic and lignocellulosic composition [56, 57]. In a previous study, Wang et al. [58] demonstrated the use of wine residue as a carbon precursor, chemically activated with ZnCl_2_ in the presence of melamine and FeCl_3_ as N and Fe sources, respectively. The resulting catalyst exhibited a half‐wave potential of 0.84 V versus reversible hydrogen electrode (RHE) for the ORR in alkaline media (0.1 M KOH), which was attributed to its high specific surface area and the presence of N and iron dopants, enabling efficient four‐electron selectivity [58].

The present work explores the use of WP provided by *Industrias Vínicas S.A.*, a company that recycles and reuses solid biomass waste from vineyard operations in the central zone in Chile [59]. Here, we investigate how KOH and ZnCl_2_ modulate the structural evolution, active‐site formation, and ORR performance of Fe‐N‐C materials produced via one‐step synthesis without the use of nitrogen precursors. XPS analysis confirmed that WP undergoes a nitrogen self‐doping process during pyrolysis, leading to the formation of N‐functionalities and Fe‐N_*x*_ active sites. Notably, a higher specific surface area was obtained with KOH activation compared to ZnCl_2_. For the KOH‐treated WP with Fe, enhanced ORR electrocatalytic activity was observed, achieving onset potentials of 0.87 V_RHE_ (@0.1 mA cm^−2^) for ORR. We propose that KOH activation, compared to ZnCl_2_, promotes the development of electrochemically accessible mesopores and, consequently, a greater utilization of Fe‐N_x_ sites, thereby enhancing both the electrocatalytic activity and the 4e^−^ selectivity of the ORR in alkaline media. In contrast, ZnCl_2_ tends to collapse part of the network after acid washing, thus hampering the effective electrochemically active surface area (ECSA) and selectivity. To the best of our knowledge, this is the first systematic investigation employing different one‐step activating reagents for the conversion of WP into porous, iron‐based carbonaceous ORR catalysts, without the addition of an external nitrogen source.

## 2 Results and Discussion

### 2.1 Thermal Decomposition Behavior

Biomass‐derived Fe‐N‐C catalysts were obtained using WP as carbon–nitrogen precursor, in combination with an iron source and a chemical activating agent. KOH or ZnCl_2_ were employed as an activating agents to tailor the structural and chemical properties of the resulting materials. The mixtures were subjected to pyrolysis under an inert N_2_ atmosphere, promoting the formation of catalytically active Fe‐N_x_ sites, which are known to facilitate the ORR [12, 60, 61, 62, 63]. This thermal treatment also contributed to the development of a porous carbon structure, enhancing the specific surface area and overall catalytic performance of the final materials [37, 38, 39, 42, 43].

**FIGURE 1:**
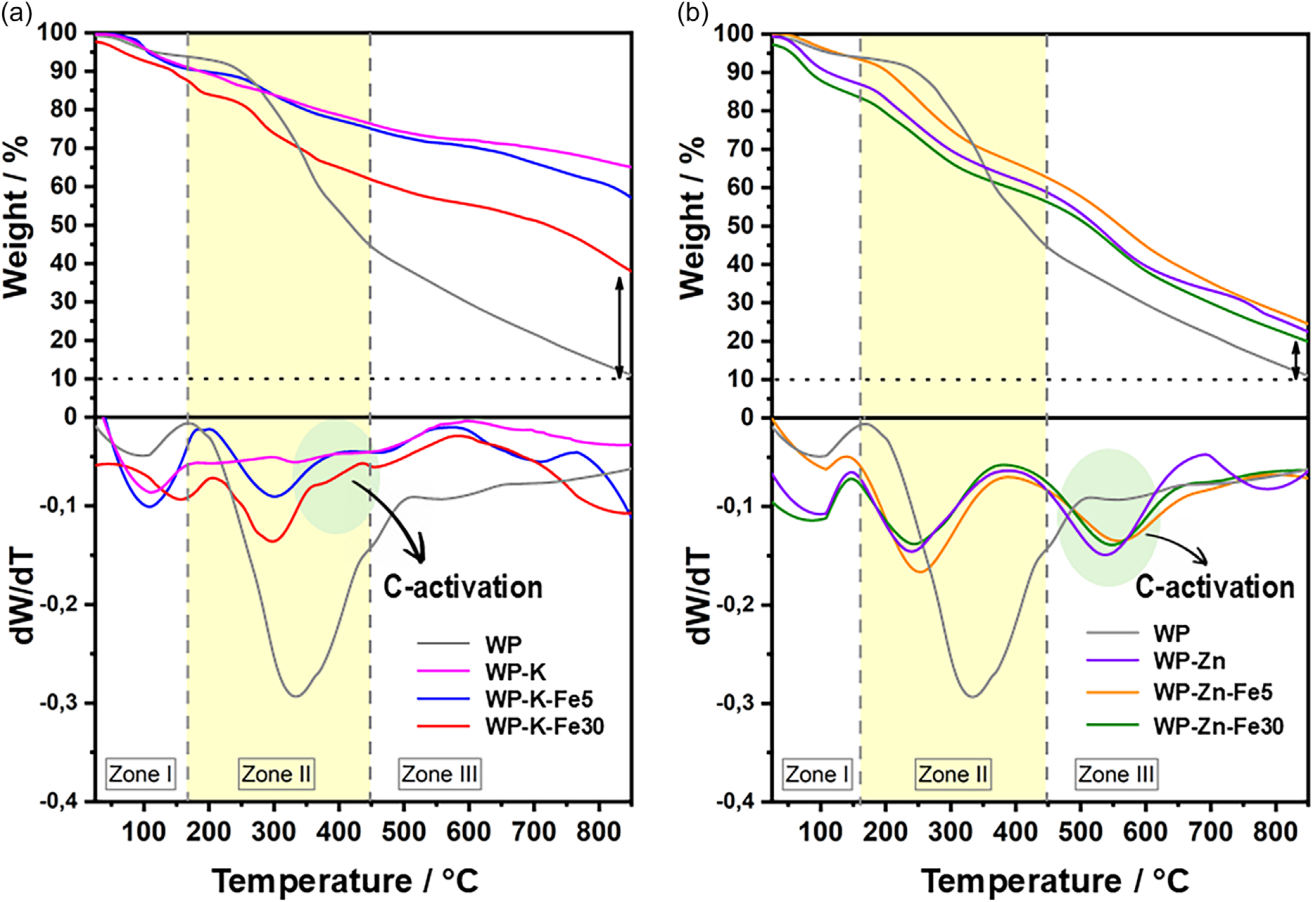
TGA and DTG (dW/dT) curves of raw WP (gray) and: (a) WP‐K (pink), WP‐K‐Fe5 (blue), and WP‐K‐Fe30 (red); (b) WP‐Zn (purple), WP‐Zn‐Fe5 (orange), and WP‐Zn‐Fe30 (green).

Prior to chemical activation, the WP was pretreated by washing and drying to remove soluble components such as residual sugars, phenolic compounds, minerals, and pigments [57, 64]. Elemental analysis of the treated WP revealed an organic composition of C 49.89, N 2.20, H 6.52, and S 0.255 wt%. The activation process was conducted using a single‐step procedure in which the pretreated WP was wet‐impregnated with iron salt (5 and 30 wt.%) and either KOH or ZnCl_2_. The impregnated biomass was then pyrolyzed at 850°C with a heating rate of 5°C min^−1^ (see Experimental section). Postpyrolysis, the resulting composites were leached with 0.5 M H_2_SO_4_ solution to remove residual activating agents and unanchored metallic nanoparticles formed during the thermal treatment [12, 15, 38, 40, 46]. The thermal decomposition behavior of the raw WP and WP‐treated composites prior to pyrolysis (WP‐K, WP‐K‐Fe5, WP‐K‐Fe30, WP‐Zn, WP‐Zn‐Fe5, and WP‐Zn‐Fe30) was evaluated by thermogravimetric analysis (TGA), as shown in Figure 1a,b. The TGA reveals that *c.a.* 90% of the untreated WP mass is lost at 850°C (gray line, Figure 1, Table S1), indicating extensive thermal degradation. The derivative thermogravimetric curves (DTG, dW/dT) show three distinct decomposition zones. Zone I (from room temperature to ~170°C) corresponds to the evaporation of physically adsorbed moisture. Zone II (up to ~450°C) is attributed to the thermal decomposition of cellulose and hemicellulose components [65, 66, 67]. Finally, zone III (450 – 850°C) reflects the breakdown of lignocellulosic structures and the gradual formation of a thermally stable carbonaceous char [46, 65, 68].

Significant differences are observed in the thermal decomposition of WP samples impregnated with activating agents (KOH or ZnCl_2_) and Fe salt compared to raw WP (Figure 1a,b). According to the literature, KOH is frequently employed in two‐step biomass activation processes, as its strong alkalinity promotes the hydrolysis of glycosidic bonds in cellulose and hemicellulose, as well as the cleavage of ether and ester bonds in lignin. These reactions lead to partial depolymerization and solubilization of the biomass matrix [38, 41]. This effect is clearly evidenced in the DTG curve of WP‐K (pink line, Figure 1a), where the characteristic decomposition peaks associated with hemicellulose (220‐315°C), cellulose, and lignocellulose (both above 315°C), which correspond to zones II and III, are almost completely suppressed [65, 69]. This disappearance suggests that substantial degradation of the biomass components has already occurred during the chemical impregnation stage prior to pyrolysis. Interestingly, when Fe salts are introduced into the activation mixture (WP‐K‐Fe5 and WP‐K‐Fe30), the peaks in zones II and III persist, indicating a moderated decomposition process. Such behavior can be rationalized by considering the formation of iron hydroxide species in alkaline media. Specifically, Fe^2+^ reacts with OH^‐^ ions to form Fe(OH)_2_ (Equation (1), *Kps =* 4.87 × 10^−17^), which can further convert into [Fe(OH)_4_]^2‐^ complexes under strong alkaline conditions (Equation (2) ).

(1) $${{\mathrm{Fe}}^{2}}^{+}\;+\;2{\mathrm{OH}}^{‐}\;⇄\;\mathrm{Fe}{\left(\mathrm{OH}\right)}_{2\;\left(s\right)}$$

(2) $$\mathrm{Fe}{\left(\mathrm{OH}\right)}_{2\;(s)}\;+\;2{\mathrm{OH}}^{‐}\;⇄{\left[\mathrm{Fe}{\left(\mathrm{OH}\right)}_{4}\right]}^{2‐}$$

Given the high pH induced by excess KOH during the wet‐impregnation stage, the transient formation of [Fe(OH)_4_]^2‐^ is plausible under these experimental conditions (pH ~ 14). This interpretation is supported by the final residue mass data (Table S1), where composites with higher Fe content exhibit greater residue retention compared to raw WP, indicating a reduced extent of biomass degradation during pyrolysis. In contrast, WP materials activated with ZnCl_2_ (WP‐Zn: purple line; WP‐Zn‐Fe5: orange line; WP‐Zn‐Fe30: green line; Figure 1b) exhibit a thermal decomposition profile comparable to that of raw WP (Table S1). This similarity can be attributed to the fact that ZnCl_2_, acting as a Lewis acid, is not capable of inducing the hydrolysis of glycosidic bonds in cellulose and hemicellulose, nor the cleavage of ether and ester bonds in lignin. Moreover, Fe^2+^ ions are expected to remain predominantly uncoordinated in the presence of ZnCl_2_, as the formation of iron‐chloride complexes is not favored under the conditions employed. Nevertheless, the observed peak shifts in the DTG curves for both KOH‐ and ZnCl_2_‐activated materials confirm that the presence of activating agents reduces the onset temperature for lignin thermal decomposition. This behavior is influenced by both the melting point of the activating agent and its specific interaction mechanism with the biomass during the pyrolysis process [65, 69].

### 2.2 Structural Characterization

**FIGURE 2:**
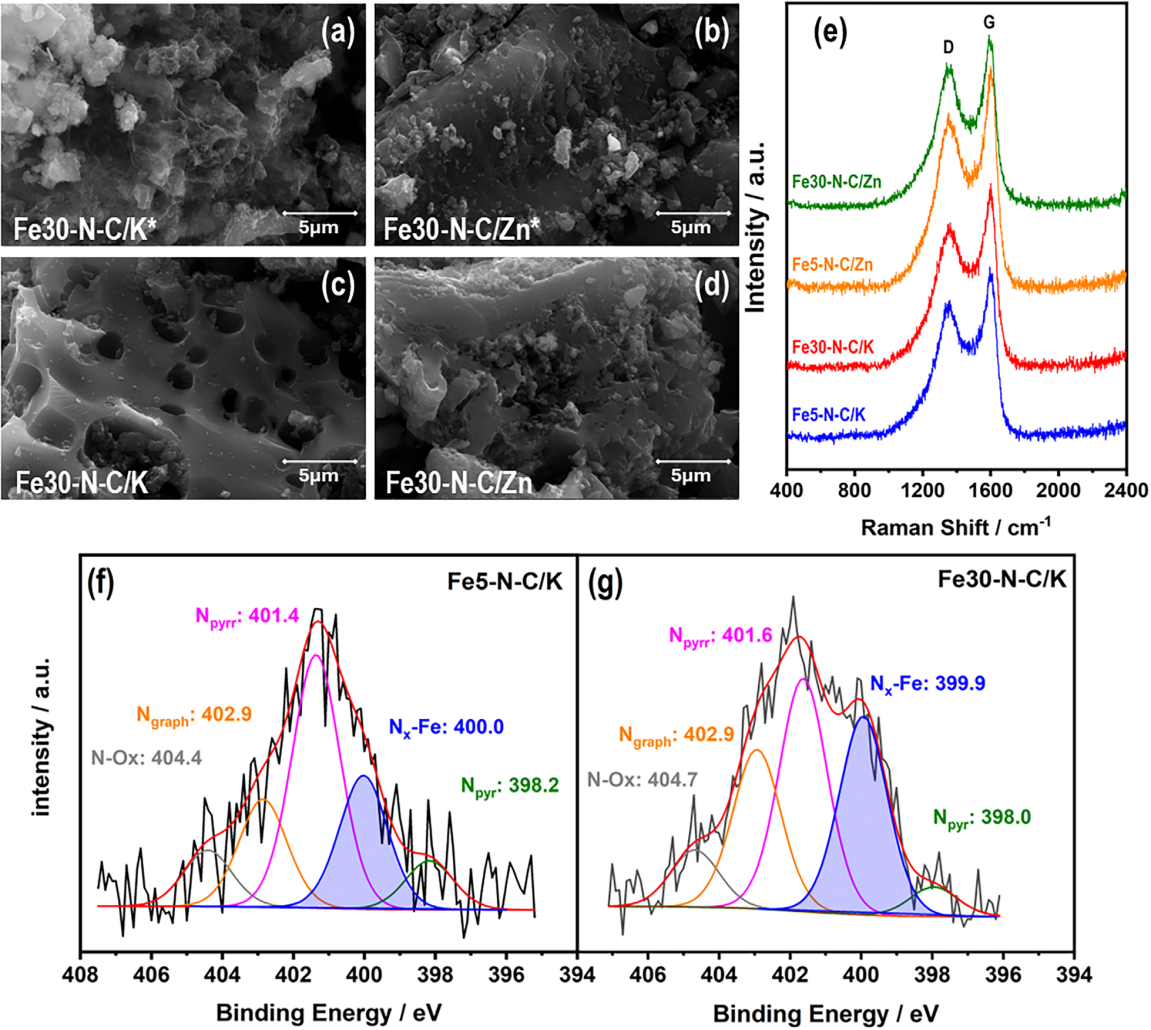
FESEM images recorded from pyrolyzed samples: (a) Fe30‐N‐C/K^*^ and (b) Fe30‐N‐C/Zn*; and lixiviated samples: (c) Fe30‐N‐C/K and (d) Fe30‐N‐C/Zn. (e) Raman spectra for Fe‐N‐C/K and Fe‐N‐C/Zn composites. XPS N 1s spectra recorded for (f) Fe5‐N‐C/K, and (g) Fe30‐N‐C/K. The highlighted blue area indicates the contribution of Fe‐N_*x*_ species.

The textural properties of Fe‐doped samples were determined using the Brunauer–Emmett–Teller (BET) method for the pyrolyzed and leached samples. As shown in Table 1, the specific surface area (*S*_BET_) of Fe‐N‐C/K samples reaches ~2000 m^2^ g^−1^ compared to the nonleached sample. Additionally, the total pore volume increases 4–5 times after the acid‐leaching treatment. This significant increase is attributed to the effective removal of remanent intercalated K species and Fe‐based nanoparticles, which previously occupied or blocked the pore network [39]. In contrast, Fe‐N‐C/Zn exhibits a significant reduction in both surface area and pore volume relative to the corresponding nonleached samples, suggesting that the acid treatment induces a pronounced structural collapse. Nevertheless, the average pore diameter remains within the mesoporous range (2–50 nm). This collapse is associated with the migration and removal of remanent Zn species in the form of ZnO and unanchored Fe nanoparticles generated during pyrolysis, which destabilizes the carbon framework [42, 70]. This difference is evidenced by FESEM analysis shown in Figures 2a‐e and S1b‐e. A noticeable increase in surface roughness is observed for KOH‐activated samples after acid leaching, which aligns with its high porosity. However, treating WP with both activating agents leads to a less compact surface and lower uniformity than raw WP (Figure S1a). In WP‐Zn, a *S*_BET_ value of 7.83 m^2^ g^−1^ and a pore volume (*V*_p_) of 0.052 cm^3^ g^−1^ are observed. The estimated average pore diameter, *d*_a_vg ≈ 4·*V*_p_/*S* (with the proper conversion from m^2^ to cm^2^), yields ~27 nm, which is consistent with large mesopores and/or interparticle voids. Consequently, a moderate pore volume can coexist with a very low specific surface area due to the absence of micro/mesopores.

**TABLE 1:** Textural properties of biomass‐derived Fe‐N‐C catalysts.

Sample	**S**_**BET**_ **,** **m** ^**2**^ **g** ^**−1**^	Pore diameter, nm	**Pore volume, cm** ^**3**^ **g** ^**−1**^
WP	5.47	6.67	0.0095
WP‐K	122.25	2.70	0.084
WP‐Zn	7.83	20.37	0.052
Fe5‐N‐C/K^*^	380.4	3.10	0.312
Fe5‐N‐C/K	1993.2	2.07	1.046
Fe30–N‐C/K^*^	400.9	2.68	0.280
Fe30–N‐C/K	2092.3	2.59	1.398
Fe5‐N‐C/Zn^a^	817.9	1.67	0.343
Fe5‐N‐C/Zn	498.9	1.68	0.210
Fe30‐N‐C/Zn^a^	613.7	2.21	0.440
Fe30‐N‐C/Zn	467.3	2.15	0.253

a Samples, before leaching.

The graphitization degree of Fe‐doped samples was analyzed by Raman spectroscopy through the characteristic D and G bands. Figure 2e shows the Raman spectra of the acid‐leached samples. All spectra exhibit broadened and overlapped D (~1350 cm^−1^) and G (~1585 cm^−1^) bands. Table S2 summarizes both intensity and area ratios (D/G) for Fe‐N‐C/K and Fe‐N‐C/Zn samples, where higher values are indicative of lower graphitization degrees [25, 71]. The calculated intensity ratios are *c.a.* 0.8, and the area ratios range from 2.3 to 2.7, indicating that the carbon is partially ordered in all samples [71, 72]. This order arises from the Fe content, which promotes graphitization through a homogeneous solid‐state catalytic mechanism [73]. Among them, the Fe30‐N‐C/K displays the highest A_D_/A_G_ ratio. In addition, this sample also shows enhanced surface roughness (Figure 2c) and the highest specific surface area, with a mesopore diameter of 2.6 nm and a total pore volume of 1.4 cm^3^ g^−1^ (Table 1 and Figure 2). These parameters suggest a favorable combination of defect density and accessible porosity, which is expected to boost its ORR electrocatalytic response.

The surface chemical composition of the synthesized materials was studied by XPS analysis. Figure 2f‐g shows the N 1s XPS spectra recorded for KOH‐activated biomass with 5 and 30 wt.% Fe, respectively. As can be observed, the N 1s spectra indicate the presence of various nitrogen functionalities, including pyridinic, pyrrolic, graphitic, and oxidized nitrogen species, together with contributions commonly assigned to Fe‐N_*x*_ species. These functionalities were found in all four leached materials. For comparison, the N 1s spectra of Fe‐N‐C/Zn samples are included in Figure S2. The presence of contributions associated with Fe‐N_*x*_ coordination suggests the formation of Fe‐N‐C‐type structures derived from WP. In the literature, Fe‐N_*x*_ moieties have been widely associated with enhanced ORR activity and selectivity, although contributions from other species such as nitrogen functionalities and carbon defects have also been reported [6, 12, 13, 21, 25, 60, 61, 62, 74, 75, 76, 77, 78, 79, 80]. The deconvolution of the N 1s spectra provides insights into the evolution of surface nitrogen species as a function of the synthetic conditions. As is observed in Table S3, increasing the Fe content from 5 to 30 wt% is associated with a moderate increase in the relative concentration of Fe‐N_*x*_ species (from ca. 20% to ca. 30%), along with a decrease in pyrrolic‐N. Notably, more pronounced differences are observed in the distribution of N functionalities depending on the activating agent. ZnCl_2_‐activated samples exhibit higher contributions of graphitic and oxidized nitrogen species, while KOH‐activated samples show lower levels of oxidized nitrogen. These differences are expected to influence the electronic properties of the carbon matrix and, consequently, the ORR performance. Such variations in surface compositions highlight the complex interplay between Fe‐N_*x*_ species, N functionalities, and the carbon structure.

### 2.3 Electrochemical Characterization and Electrocatalytic Studies

The synthesized WP‐derived materials were evaluated as potential electrocatalysts for the ORR in alkaline media. Before the electrocatalytic studies, the ECSA of the samples was determined by cyclic voltammetry at different scan rates (Figure S3). The ECSA values were obtained from the slope of Δ*i* as a function of the scan rate (Figure S4), divided by the specific capacitance (*C*_s_) of carbonaceous materials (see Experimental section), and the values are summarized in Table 2. As can be observed, the WP carbon samples exhibited similar ECSA values regardless of the chemical activation method used. However, the iron‐containing samples showed an increase in the double‐layer capacitance (*C*_dl_), which correlates with higher ECSA, particularly when KOH was employed as the activating agent.

**TABLE 2:** Electrochemical and ORR parameters for WP‐derived electrodes.

Sample	**ECSA / cm** ^**2**^	***E***_**onset**_ **/ V versus RHE**	*n*	**Tafel slope / V dec** ^**−1**^
WP‐K	13.1	0.73 ± 0.01	2.8 ± 0.1	−0.070 ± 0.002
Fe5‐N‐C/K	149.8	0.83 ± 0.03	3.5 ± 0.2	−0.058 ± 0.003
Fe30‐N‐C/K	282.0	0.87 ± 0.02	4.0 ± 0.1	−0.069 ± 0.001
WP‐Zn	18.7	0.74 ± 0.03	2.9 ± 0.1	−0.077 ± 0.004
Fe5‐N‐C/Zn	54.4	0.78 ± 0.02	2.9 ± 0.2	−0.101 ± 0.003
Fe30‐N‐C/Zn	118.2	0.81 ± 0.02	3.6 ± 0.3	−0.116 ± 0.005

**FIGURE 3:**
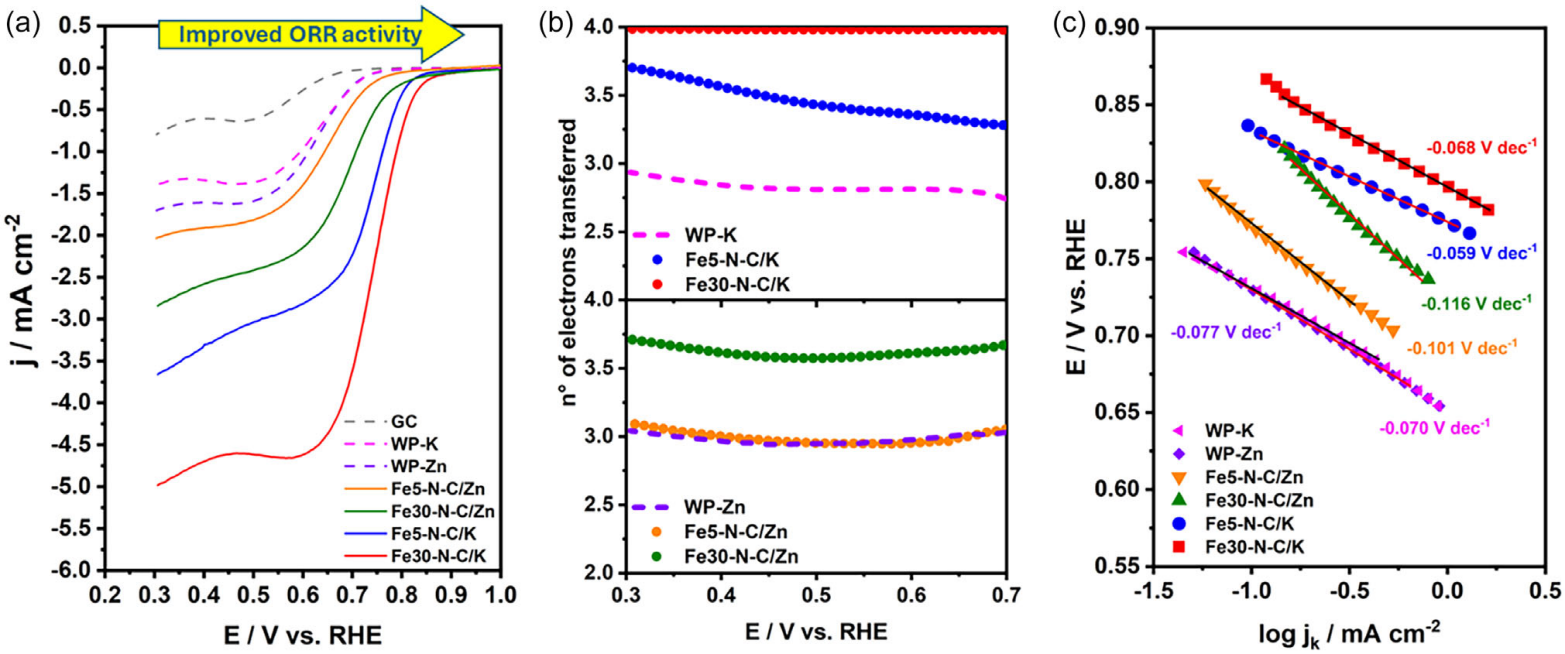
(a) Polarization curves for ORR on WP‐derived catalyst‐modified electrodes in 0.1 M KOH solution saturated with O_2_ (scan rate: 5 mVs^−1^, rotation rate: 1600 rpm). (b) Number of electrons transferred during the ORR as a function of the applied potential. (c) Tafel plots for the ORR on catalyst‐modified electrodes.

The ORR was evaluated by linear sweep voltammetry (LSV) in O_2_‐saturated alkaline media, as shown in Figure 3, and the main electrochemical parameters are summarized in Table 2. The onset potential (*E*_onset_) is considered the activity descriptor, since it reflects the overpotential (or energy) required to activate the oxygen molecule. As observed in Figure 3a, all catalyst materials exhibit electrocatalytic activity toward ORR compared to a bare glassy carbon (GC) electrode. The metal‐free C–N samples (dashed lines, WP‐K and WP‐Zn) exhibit similar onset potentials, suggesting that the activating agent alone does not determine the ORR activity. These results also indicate that carbon defects and nitrogen functionalities serve as intrinsic active sites, albeit with lower activity than Fe‐containing catalysts [5, 81]. In contrast, iron‐doped catalysts (solid lines) show more positive *E*_onset_ than their metal‐free counterparts (dashed lines), which is consistent with the contribution of Fe‐based active sites within the graphitized carbon matrix. The presence of Fe‐N_x_ species is supported by XPS analysis (Figures 2f‐g and S2a,b), although their exact coordination environment cannot be conclusively determined by this technique alone. Similar trends have been reported for other Fe‐doped biomass‐derived catalysts in the literature (Table 3) [15, 58, 81]. In addition, a clear improvement is observed for the KOH‐activated samples, suggesting that this activation route enhances the exposure and accessibility of active sites.

**TABLE 3:** Electrochemical parameters for ORR in alkaline conditions (0.1 M KOH) of Fe‐N‐C materials derived from different biomass sources.

Biomass source	Activating agent	Additional source		Pyrolysis process	***E***_**onset**_ **/ V versus RHE**	***E***_**1/2**_ **/ V versus RHE**	*n*	**Tafel slope** **mV dec** ^**−1**^	Ref.
Soybean straw	MgO		Melamine	One‐step	0.98	0.85	3.97	69	[75]
Spinach	NaCl / KCl		Melamine	Two‐step	–	0.88	3.91	61	[82]
Waste wine	ZnCl_2_		Melamine	Two‐step	–	0.84	3.70	–	[58]
Soybean dregs			g‐C_3_N_4_	One‐step	0.89	–	3.59	80	[83]
Corn stalks	–		Melamine	One‐step	0.99	0.85	3.72	–	[76]
Wood	–		Urea	Two‐step	0.98	0.9	3.9	80	[77]
Sengon wood	–		PANI	Two‐step	0.84	0.79	3.99	85	[84]
Pig blood	–		–	Two‐step	0.999	0.875	3.78	–	[85]
Black fungus	ZnCl_2_		Melamine	One‐step	–	0.896	–	78	[78]
Acorn shell	Mg_5_(OH)_2_(CO_3_)_4_		Melamine	Two‐step	0.99	0.84	3.92	88	[79]
Auricularia auricular‐judae	–		Urea	One‐step	1.0	0.89	3.99	59.3	[86]
Coffee grounds	–		NH_3_	One‐step	–	0.87	3.92	129	[80]
Spirulina	K_2_CO_3_		Urea / NH_4_Cl	Two‐step	0.99	0.86	3.66	–	[87]
Biogas residue	KOH		Melamine/1,10‐phen	Two‐step	0.96	0.74	3.83	–	[88]

To rationalize this trend, the *E*_onset_ was correlated with the electrochemical double‐layer capacitance (*C*_dl_), used as a proxy for the ECSA. As shown in Figure S5a, a positive correlation between the *C*_dl_ and *E*_onset_ is observed, whereas no clear linear trend is found when *E*_onset_ is compared with the BET surface area. This discrepancy reveals that not all the surface area probed by N_2_ physisorption is accessible to the electrolyte. Indeed, part of the BET surface area corresponds to micropores that cannot accommodate solvated ions. Instead, the effective electrochemical activity is governed by the development of mesopores. This interpretation is further supported by Figure S5b, which shows a linear relationship between *C*_dl_ and the average pore diameter, where samples with larger mesopores (>2 nm) exhibit higher *C*_dl_ values, indicating enhanced accessibility of the electrolyte to the internal carbon surface. Therefore, the catalytic enhancement observed for Fe‐N‐C/K can be attributed not only to the presence of Fe‐N_x_ sites but also to the favorable pore architecture generated by KOH activation, which maximizes the electrochemical utilization of active sites. In addition, the activity of Fe‐N_x_ sites is expected to be influenced by the local carbon environment, including nitrogen functionalities, structural defects, and possibly formed iron‐based nanoparticles, which can modulate the electronic structure of the active sites and facilitate charge transfer during ORR [60, 61, 74, 75, 76, 77, 78, 79]. Interestingly, even a Fe content as low as 5% in the presence of KOH achieves a remarkable electrocatalytic activity, highlighting the potential to deliver efficient performance with minimal iron loading.

To further probe the presence and electrocatalytic role of Fe‐N_x_ sites in these systems, LSV measurements were performed in 0.1 M KOH solution containing 10 mM KCN, which is a well‐known poison for Fe‐coordinated active sites [12]. As shown in Figure S6, a noticeable negative shift in the polarization curves (*ca.* 100 mV) and a decrease in the limiting current density are observed after cyanide addition, indicating a loss of ORR activity compared to the pristine samples. This electrocatalytic behavior has been reported in the literature as indicative of the involvement of Fe‐N_*x*_ moieties in the ORR, due to strong coordination of CN^‐^ ions to Fe centers, which blocks their electrocatalytic functions [12, 62]. These results provide complementary electrochemical evidence, supporting the contribution of Fe‐based active sites, in agreement with the XPS analysis.

The changes observed in the mass‐transfer limited current densities during the ORR LSV test (Figure 3a) could be associated with the mechanism and selectivity of the reaction. The ORR can proceed via either a two‐electron pathway, producing hydrogen peroxide as an intermediate product, or a more desirable four‐electron pathway, leading to the direct formation of water or OH^‐^ in alkaline conditions. The two‐electron route is less favorable, since the hydrogen peroxide can degrade the carbon support of the catalysts, membranes, and other components in energy conversion devices [89]. Figure 3b shows the total number of electrons transferred during the ORR for the different Fe‐N‐C and metal‐free C–N catalysts. The results reveal that the Fe30‐N‐C/K catalyst predominantly follows a four‐electron pathway, as further confirmed by the rotating ring‐disk electrode measurements (Figure S7). In contrast, the other systems exhibit a lower average electron transfer number, indicating that the ORR proceeds via a mixed 2e^‐^/4e^‐^ pathway. This behavior is accompanied by a higher peroxide yield compared to the Fe30‐N‐C/K sample (Figure S7). Interestingly, the Fe5‐N‐C/Zn catalyst exhibits similar selectivity behavior to the WP/Zn sample. In both metal‐free WP/Zn and WP/K samples, the selectivity toward the four‐electron pathway is limited, producing a higher percentage yield of hydrogen peroxide during the catalytic process, as expected for matrices where nitrogen functionalities and/or carbon defects act solely as the primary ORR active sites [5, 81].

To understand the mechanism and corroborate the activity performance, a Tafel slope analysis has been conducted, as shown in Figure 3c. Tafel slopes were obtained in the kinetic region of the LSV curves, avoiding the influence of diffusional currents. As can be seen, there is a change in the Tafel slope values depending on the activation method; the Fe/KOH‐activated samples present lower Tafel slopes close to the theoretical value of −0.059 V  dec^−1^, which indicates better electrocatalytic activity. Based on these values and on the evidence that the Fe‐N_*x*_ moieties could be acting as the principal active sites in the prepared catalysts, in accordance with the XPS analysis and cyanide poisoning test, the following mechanism could be proposed (Equations (3) and (4) ). In this case, the rate‐determining step corresponds to the adsorption of O_2_ in the Fe‐N_x_ sites (Equation (4) ) [74]:

(3) $${\left[\mathrm{Fe}(\mathrm{III})‐{\mathrm{OH}}^{‐}\right]}_{\mathrm{ad}}\;+\;H_{2}O\;+\;e^{‐}\;⇄\;{\left[\mathrm{Fe}(\mathrm{II})‐{\mathrm{OH}}_{2}\right]}_{\mathrm{ad}}\;+\;{\mathrm{OH}}^{‐}$$

(4) $${\left[\mathrm{Fe}(\mathrm{II})‐{\mathrm{OH}}_{2}\right]}_{\mathrm{ad}}\;+\;O_{2}\;\to \;{{\left[\mathrm{Fe}(\mathrm{III})‐O_{2}\right]}^{‐}}_{\mathrm{ad}}\;+\;H_{2}O\;⇄\;{\left[\mathrm{Fe}(\mathrm{II})‐O_{2}\right]}_{\mathrm{ad}}\;+\;H_{2}O\;(r.d.s.)$$

This mechanism resembles the previously proposed one for the Fe‐N‐C electrocatalysts in acid and alkaline media [74]. The Tafel slope values determined for the Fe/ZnCl_2_‐activated materials are higher than those of the Fe/KOH‐activated catalysts, corroborating the lower activity when ZnCl_2_ is used as an activator. The higher Tafel slope indicates a change in the reaction mechanism of these materials, with Equation (5) representing the rate‐determining step*.*

(5) $${\left[M(\mathrm{II})‐{\mathrm{OH}}_{2}\right]}_{\mathrm{ad}}\;+\;O_{2}\;+\;e^{‐}\;⇄\;{{\left[M(\mathrm{II})‐O_{2}\right]}^{‐}}_{\mathrm{ad}}\;+\;H_{2}O\;(r.d.s.)$$

For comparison purposes, Table 3 summarizes the electrocatalytic parameters reported in the literature for Fe‐N‐C materials derived from different biomass sources. The electrocatalytic performance of Fe‐N‐C/K catalysts, in terms of onset potential, number of transferred electrons, and Tafel slope, falls within the range of previously reported biomass‐derived catalysts. Nevertheless, these values are achieved in a one‐step pyrolysis process without the addition of an external nitrogen source and with a minimal Fe loading.

These results demonstrate that the chemical nature of activating agents strongly influences the electrochemical behavior of WP‐derived catalysts. The activation route based on KOH combined with Fe enhances the ORR electrocatalytic activity, as reflected by improved onset potentials and higher selectivity. In contrast, activation with ZnCl_2_ leads to structural collapse after leaching, which limits the electrocatalytic performance toward ORR. The differences revealed by textural and electrochemical characterizations indicate that the ECSA, double‐layer capacitance, and mesopore development are closely linked to the ORR performance. Nevertheless, establishing a general correlation between these parameters and the electrocatalytic activity of biomass‐derived catalysts will require additional systematic studies. To the best of our knowledge, this work represents the first systematic comparison of different activation strategies applied to wine pomace‐derived Fe‐N‐C catalysts, laying the groundwork for the valorization of wine industry waste by transforming it into value‐added electrocatalytic materials for energy conversion devices.

## 3 Conclusions

This study highlights the potential of wine industry biowaste as a sustainable precursor for the synthesis of efficient Fe‐N‐C electrocatalysts for ORR. The results reveal that the choice of activating agent strongly influences the thermal decomposition pathway, which in turn determines the textural, structural, and electrochemical properties, as well as the overall electrocatalytic ORR activity of the materials. Structural characterization suggests that KOH activation enhances surface roughness and improves electrolyte accessibility to the Fe‐based active sites. In contrast, ZnCl_2_ activation generates a porous structure but promotes instability of the carbon matrix upon acid leaching, leading to partial pore collapse and consequent decrease in electrocatalytic activity. The Fe‐N‐C catalysts activated with KOH exhibit the highest ORR activity and selectivity, predominantly following a four‐electron pathway. In contrast, the ZnCl_2_‐activated catalysts display a mixed reaction pathway, accompanied by increased peroxide formation. Overall, enhanced electrocatalytic activity is attributed to the combined effect of Fe‐N_*x*_ species, favorable pore architecture, and local carbon environment, including nitrogen functionalities and structural defects, which together govern the accessibility and intrinsic activity of the active sites.

Finally, this study demonstrates that the one‐step pyrolysis of wine pomace with KOH and Fe, without the need for an external nitrogen source, enables the synthesis of Fe‐N‐C catalysts with strong potential for electrochemical energy conversion applications. This approach highlights the effective valorization of winemaking biowaste into high‐value functional materials and reinforces the role of biomass as a sustainable and scalable precursor for next‐generation energy conversion systems.

## 4 Experimental Section

### 4.1 Synthesis of Biomass‐Derived Fe‐N‐C Catalysts

#### 4.1.1 Pretreatment of WP

In the first stage, WP, obtained from Industrias Vínicas S.A., was washed in a water bath at 60 °C to remove soluble compounds, dried overnight at 80°C, and then pulverized. The resulting powder was used as the carbon precursor for catalyst synthesis.

#### 4.1.2 Synthesis of KOH‐activated Fe‐N‐C catalysts (Fe‐N‐C/K)

A total of 1.0 g of pulverized WP was suspended in 30 mL of Milli‐Q water under magnetic stirring. The Fe source, iron (II) tetrafluoroborate hexahydrate (Fe(BF_4_)_2_·6H_2_O, 87% purity, Sigma–Aldrich), was added at two different weight ratios relative to the carbon source (5 and 30 wt%). Potassium hydroxide (KOH, ≤100 purity, Merck) was subsequently introduced as the chemical activating agent. The mixtures were stirred for 24 h at room temperature, followed by centrifugation at 5000 rpm for 5 min. The recovered solids were dried overnight at 60 °C and then pyrolyzed at 850 °C under a nitrogen atmosphere, using a heating rate of 5°C min^−1^. The pyrolyzed materials were subsequently subjected to acid leaching in 0.5 M sulfuric acid (H_2_SO_4_, 98% purity, Merck) at 80°C. After acid leaching, the prepared materials were thoroughly washed with deionized water until a neutral pH was reached and then dried at 60°C, yielding the final catalysts Fe5‐N‐C/K and Fe30‐N‐C/K, corresponding to 5 and 30 wt% Fe loading, respectively.

#### 4.1.3 Synthesis of ZnCl_2_‐activated Fe‐N‐C Catalysts (Fe‐N‐C/Zn)

The same procedure described for the Fe‐N‐C/K catalysts was applied to this series, with the only difference being the use of zinc chloride (ZnCl_2_, p.a., Merck) as the activating agent. After leaching and washing to neutral pH, the corresponding materials were obtained: Fe5‐N‐C/Zn and Fe30‐N‐C/Zn.

For comparison purposes, metal‐free KOH‐ and ZnCl_2_‐activated WP (denoted as WP‐K and WP‐Zn, respectively) were prepared following the same procedure described above, without the addition of metal precursors. Additionally, a WP sample was prepared without any activating agents.

### 4.2 Characterization

#### 4.2.1 Elemental Analysis

The elemental composition of the raw WP was determined using a LECO CHNS‐932 elemental analyzer.

#### 4.2.2 TGA

The thermal decomposition behavior of WP was studied by TGA using a TGA 4000 system (Perkin Elmer). The analyzed samples included raw WP, metal‐free activated WP (WP‐K and WP‐Zn), and the Fe‐impregnated mixtures prior to pyrolysis (denoted as WP‐K‐Fe5, WP‐K/Fe‐30, WP‐Zn‐Fe5, and WP‐Zn‐Fe30). All experiments were conducted under a N_2_ atmosphere, with the temperature being ramped from 25 to 850°C at a constant heating rate of 5°C min^−1^.

#### 4.2.3 BET Surface Area Analysis

Textural properties were characterized using N_2_ adsorption/desorption isotherms at ‐196 °C, over a relative pressure range (*p*/*p*_0_) of 0–1 on a Micromeritics 3Flex instrument. Samples were degassed under vacuum at 180 °C for 8 h using a Micromeritics Smart VacPrep system before analysis. The resulting N_2_ physisorption isotherm data were employed to calculate specific surface areas using the BET model.

#### 4.2.4 Field Emission Scanning Electron Microscopy (FE‐SEM)

The morphology of the materials was examined using a FE‐SEM instrument (FEI Quanta FEG 250) equipped with an energy‐dispersive X‐ray spectroscopy detector.

#### 4.2.5 Raman Spectroscopy

Raman spectra were collected using a Renishaw InVia Reflex spectrometer equipped with a high‐sensitivity CCD detection system for precise spectral acquisition. Excitation was provided by a Cobalt solid‐state laser operating at 514.5 nm. The incident laser power was set to 15 mW to optimize signal intensity while minimizing the risk of sample damage. Each spectrum was obtained by averaging 10 individual acquisitions to enhance the signal‐to‐noise ratio.

#### 4.2.6 X‐ray Photoelectron Spectroscopy (XPS)

XPS measurements were performed on a SPECS spectrometer equipped with a Phoibos 150 MCD‐9 multichannel analyzer using a nonmonochromatic Mg Kα radiation (50 W, 1253.6 eV). Powdered samples (~10 mg) were loaded onto a stainless‐steel sample holder, and spectra were acquired at a constant pass energy of 30 eV over a 7 × 20 mm analysis area, at 25 °C under ultrahigh vacuum in the analysis chamber (below 10^−9^ mbar). Signal intensities were corrected using the spectrometer's transmission function. Peak fitting was conducted using CasaXPS software. The C 1s core‐level XPS peak was referenced to 284.5 eV, and a Shirley‐type background was subtracted from all spectra. Gaussian–Lorentzian functions were employed to deconvolute the binding energy contributions of each core level. Atomic surface concentrations were estimated from the integrated intensities of their most prominent photoelectron lines, corrected by their respective atomic sensitivity factors.

### 4.3 Electrochemical Analysis

Electrochemical studies were carried out in a conventional three‐electrode cell. The setup consisted of a GC rotating disk electrode (PINE E6R1, geometric area = 0.196 cm^2^) as the working electrode, an Ag/AgCl (in 3.0 M KCl) reference electrode (*E°* vs. NHE = 0.210 V), and a graphite rod as the counter electrode. Each catalyst was deposited onto the GC electrode by preparing a catalyst ink. The ink was obtained by dispersing 1 mg of catalyst in 1 mL of a mixture of isopropanol and deionized water (800 µL/200 µL, respectively), along with 10 µL of Nafion 117 solution (5 wt.% in a mixture of lower aliphatic alcohols and water, Sigma–Aldrich). The suspension was sonicated for 20 min to ensure homogeneity. Subsequently, 80 µL of the catalyst ink was drop‐casted onto the GC surface (loading 0.4 mg cm^−2^) and dried under a nitrogen (N_2_) flow. The role of the iron‐centers in ORR electrocatalysis was studied by an alkaline cyanide poisoning test in O_2_‐saturated 0.1 M KOH solution containing 10 mM KCN [12].

The ECSA was determined from the double‐layer capacitance (*C*_dl_), obtained by cyclic voltammetry (CV) at scan rates of 5, 10, 50 and 75 mV s^−1^, within a nonfaradaic potential window (Equation (6)). A specific capacitance (C_s_) value of 40 µF cm^−2^, typically associated with graphitized materials, was used as reference [90].

(6) $$ECSA=\frac{C_{dl}}{C_{s}}$$

where *C*_dl_ is the double‐layer capacitance estimated from the slope of the capacitive current density versus the scan rate plot, and *C*_s_ is the specific capacitance of the carbonaceous material (µF cm^−2^). The ORR performance was evaluated by LSV in O_2_‐saturated 0.1 M KOH at electrode rotation rates (*ω*) ranging from 400 to 2400 rpm, using a scan rate of 5 mV s^−1^. The LSV curves were 90% iR‐compensated. The onset potential (*E*_onset_) for the catalysts was determined from the LSV polarization curves at −0.1 mA cm^−2^.

## Supporting Information

Additional supporting information can be found online in the Supporting Information Section.

## Funding

This study was supported by Centro Integrativo de Biología y Química Aplicada (CIBQA), Facultad de Ciencias de la Salud, Universidad Bernardo O’Higgins, General Gana 1702, Región Metropolitana, Santiago 8370854, Chile Grant FONDECYT 11221073, 11241509, 1230991, 1221798, ANID Becas/Doctorado Nacional 21250728, ComFuturo/Horizon 2020 program (Marie Skłodowska‐Curie grant agreement) (Grant 10103426), Ministerio de Ciencia e Innovación (Grant PID2021‐123431OB‐I00 and PID2021‐123295NB‐I00) funded by MCIN/AEI/10.13039/501100011033 and by “ERDF A way of making Europe”, by the European Union NextGenerationEU/PRTR (F.J.R.), Estonian Research Council (Grant PRG2569), Estonian Ministry of Education and Research (Grant TK210, Centre of Excellence in Sustainable Green Hydrogen and Energy Technology).

## Conflicts of Interest

The authors declare no conflicts of interest.

## Supporting information

Supplementary Material

## Data Availability

The data that support the findings of this study are available from the corresponding author upon reasonable request.
