# Supplementary material for: Exploring the Role of Activating Agents on the Electrocatalytic Activity of Grape Pomace‐Derived Catalysts for the Oxygen Reduction Reaction
Source: Small Sci. 2026 May 13;6(5):e70300. doi: 10.1002/smsc.70300 (PMC13174575; doi:10.1002/smsc.70300)
Supplement: Supplementary file 1 — Supplementary Material [file SMSC-6-e70300-s001.pdf]

# Supporting Information

## Exploring the Role of Activating Agents on the Electrocatalytic Activity of Grape Pomace-Derived Catalysts for the Oxygen Reduction Reaction

Gloria Flores-Gómez, Gabriel Abarca, Cristian Silva, Gustavo Chacón-Rosales, José H. Zagal, Srinu Akula, Kaido Tammeveski*, F. Javier Recio, Ricardo Venegas*, and Karina Muñoz-Becerra*

Gloria Flores-Gómez, Gabriel Abarca, Cristian Silva Ricardo Venegas, and Karina Muñoz-Becerra
Centro Integrativo de Biología y Química Aplicada (CIBQA), Facultad de Ciencias de la Salud, Universidad Bernardo O'Higgins, General Gana 1702, Región Metropolitana, Santiago 8370854, Chile
E-mail: karina.munoz@ubo.cl

Gustavo Chacón-Rosales
Instituto de Tecnología Química (ITQ), Consejo Superior de Investigaciones Científicas-Universitat Politècnica de València, 46022, Valencia, Spain

José H. Zagal
Departamento de Química de los Materiales, Facultad de Química y Biología, Universidad de Santiago de Chile, Av. Libertador Bernardo O'Higgins 3363, Santiago, Estación Central, Región Metropolitana, Santiago 9170124, Chile

Srinu Akula, Kaido Tammeveski
Institute of Chemistry, University of Tartu, Ravila 14a, 50411 Tartu, Estonia
E-mail: kaido.tammeveski@ut.ee

F. Javier Recio
Departamento de Química Física Aplicada, Facultad de Ciencias, Universidad Autónoma de Madrid, C/Francisco Tomás y Valiente 7, Madrid 28049, España

Ricardo Venegas
Escuela de Tecnología Médica, Facultad de Ciencias de la Salud, Universidad Bernardo O'Higgins, General Gana 1702, Región Metropolitana, Santiago 8370854, Chile
E-mail: ricardo.venegas@ubo.cl

*Corresponding authors: kaido.tammeveski@ut.ee; ricardo.venegas@ubo.cl; karina.munoz@ubo.cl*

**Table S1.** Thermogravimetric analysis (TGA) obtained for WP-derived catalysts.

| Sample | Weight loss (%) | Weight retained (%) |
|---|---|---|
| **Raw WP** | 89.03 | 11.97 |
| **WP-K** | 34.95 | 65.05 |
| **WP-K-Fe5** | 43.03 | 56.98 |
| **WP-K-Fe30** | 62.08 | 37.92 |
| **WP-Zn** | 77.57 | 22.43 |
| **WP-Zn-Fe5** | 75.51 | 24.49 |
| **WP-Zn-Fe30** | 80.15 | 19.85 |

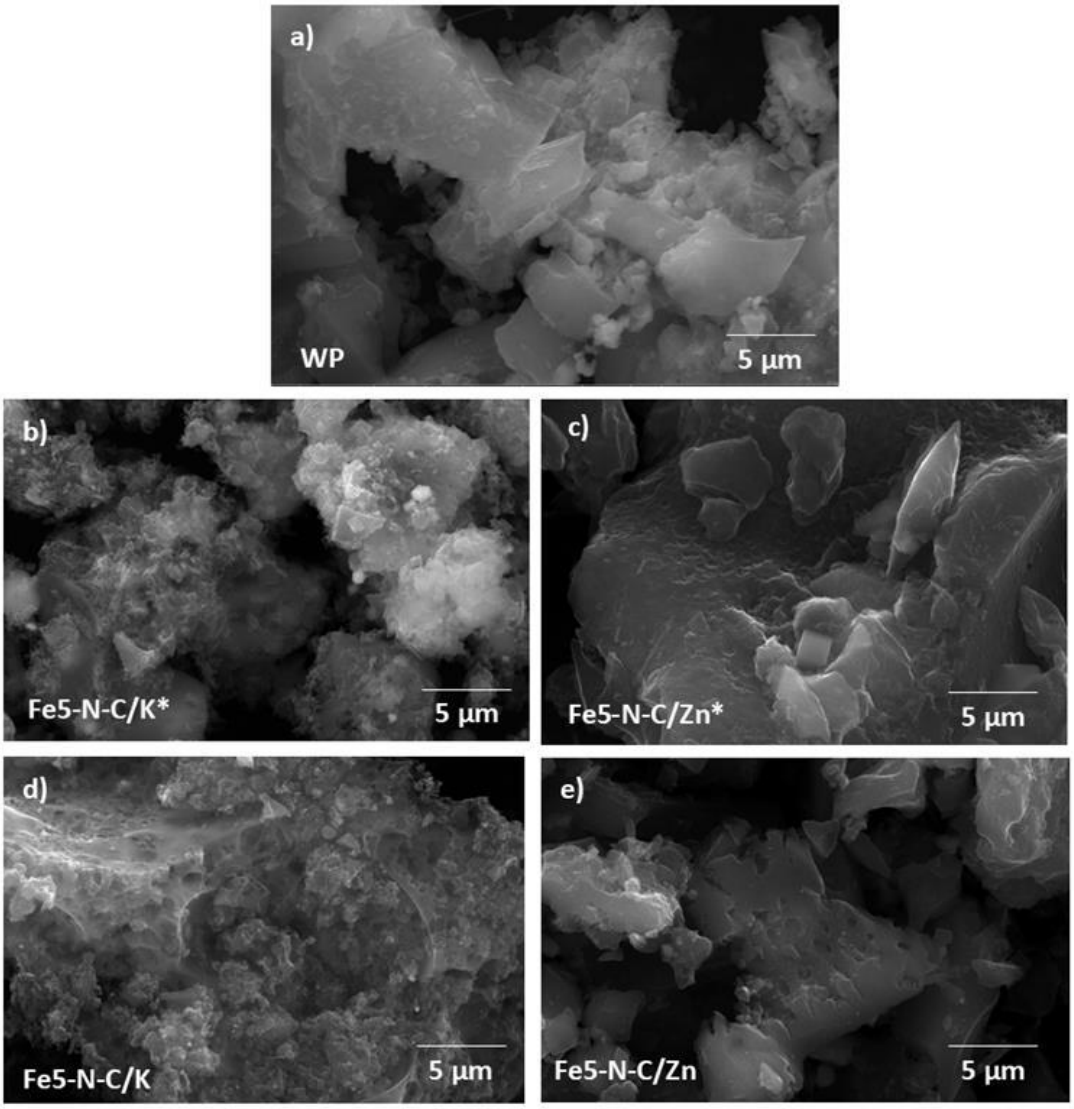


**Figure S1.** FESEM images recorded from a) WP, b) Fe5-N-C/K*, c) Fe5-N-C/Zn*, and lixiviated samples: d) Fe5-N-C/K and e) Fe5-N-C/Zn.

**Table S2.** Raman intensities and area analysis for G- and D-bands of various catalysts.

| Sample | $I_D/I_G$ | $A_D/A_G$ |
|---|---|---|
| **Fe5-N-C/K** | 0.82 $\pm$ 0.03 | 2.5 $\pm$ 0.3 |
| **Fe30-N-C/K** | 0.80 $\pm$ 0.02 | 2.7 $\pm$ 0.2 |
| **Fe5-N-C/Zn** | 0.80 $\pm$ 0.01 | 2.4 $\pm$ 0.1 |
| **Fe30-N-C/Zn** | 0.81 $\pm$ 0.03 | 2.3 $\pm$ 0.2 |

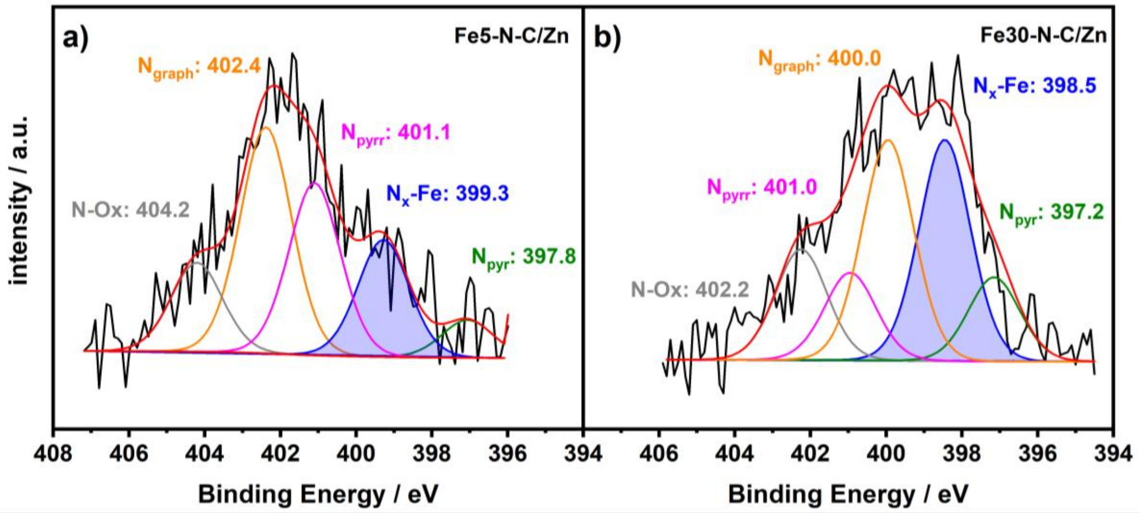


**Figure S2.** XPS N 1s spectra recorded for a) Fe5-N-C/Zn, and b) Fe30-N-C/Zn samples. The highlighted blue area indicates the contribution of Fe-$N_x$ species.

**Table S3.** Relative areas (%) of surface nitrogen species obtained from the fit of N1s XPS spectra for WP-derived catalysts.

| Sample | Npyr | Fe-Nx | Npyrr | Ngraph | N-ox |
|---|---|---|---|---|---|
| **Fe5-N-C/K** | 8.17 | 22.34 | 42.24 | 17.93 | 9.32 |
| **Fe30-N-C/K** | 4.37 | 29.39 | 34.41 | 23.47 | 8.37 |
| **Fe5-N-C/Zn** | 3.41 | 18.19 | 27.30 | 36.37 | 14.73 |
| **Fe30-N-C/Zn** | 11.66 | 30.63 | 12.05 | 30.45 | 15.21 |

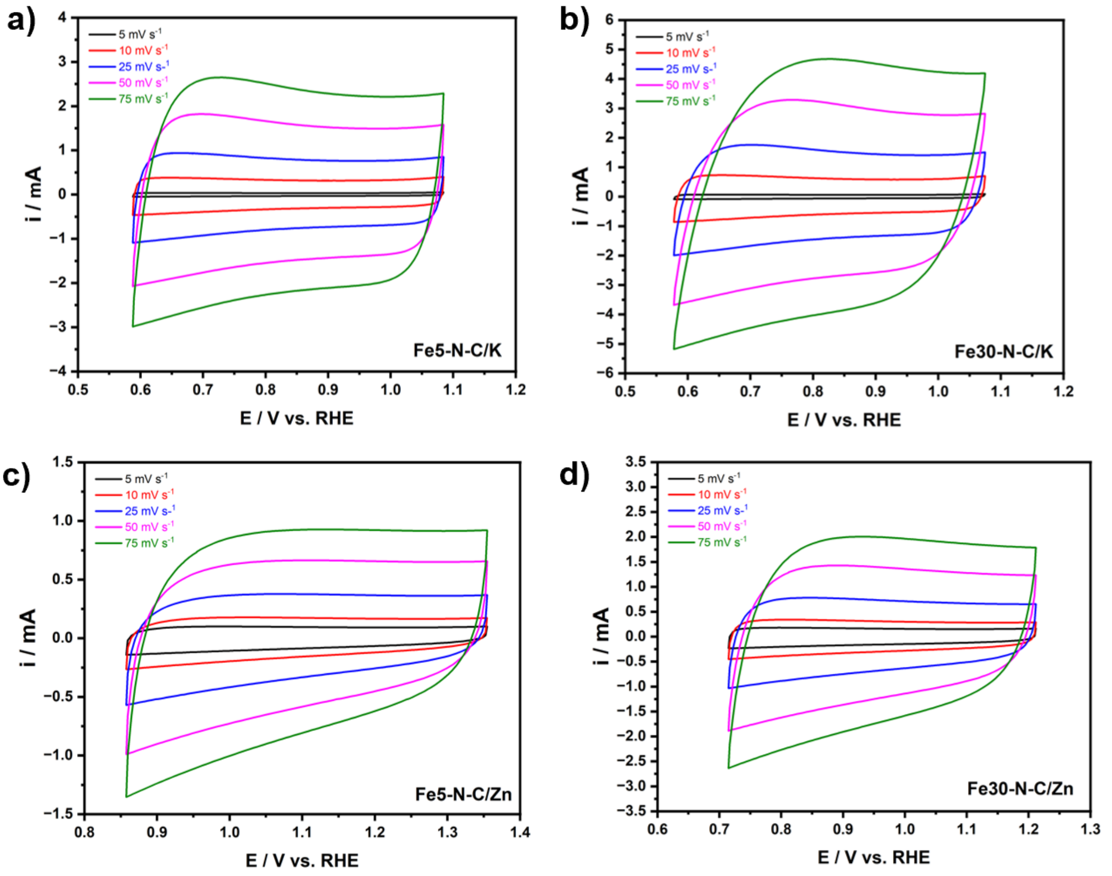


**Figure S3**. Cyclic voltammograms of catalyst-coated GC electrodes in $N_2$-saturated 0.1 M KOH solution at different scan rates for a) Fe5-N-C/K, b) Fe30-N-C/K, c) Fe5-N-C/Zn, and d) Fe30-N-C/Zn catalysts.

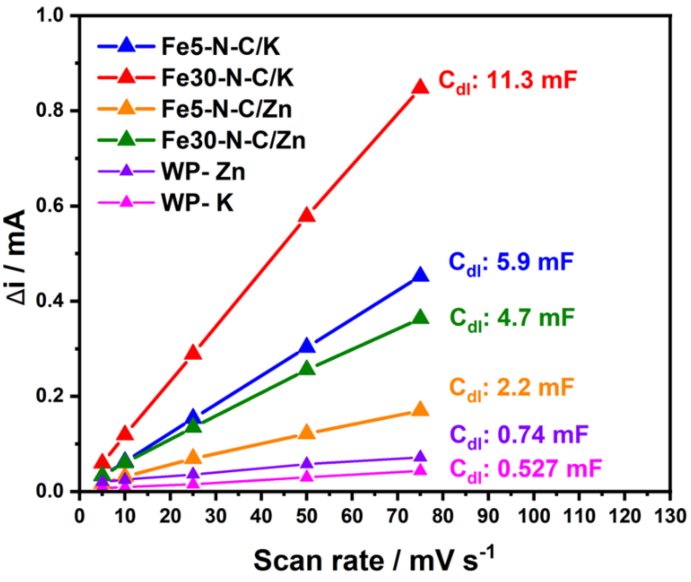


**Figure S4.** Electrochemical double-layer capacity ($C_{dl}$) of WP-K/Zn, Fe-N-C/K, and Fe-N-C/Zn catalysts.

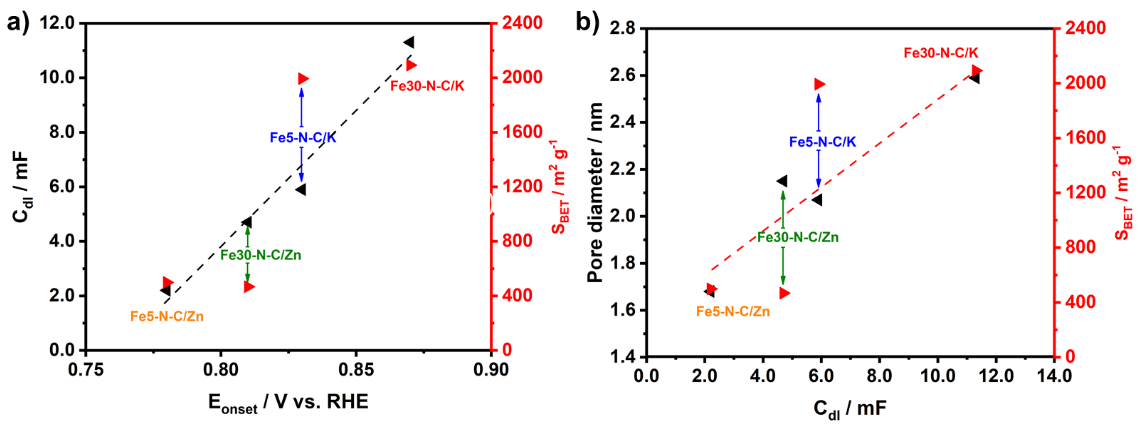


**Figure S5.** Correlation between a) the ORR onset potential and double-layer capacitance and surface BET area for WP-derived catalysts, b) the double-layer capacitance and pore diameter of the synthesized catalysts and BET surface area. KOH-activated samples exhibit higher accessibility and superior ORR activity compared with $ZnCl_2$-activated analogues.

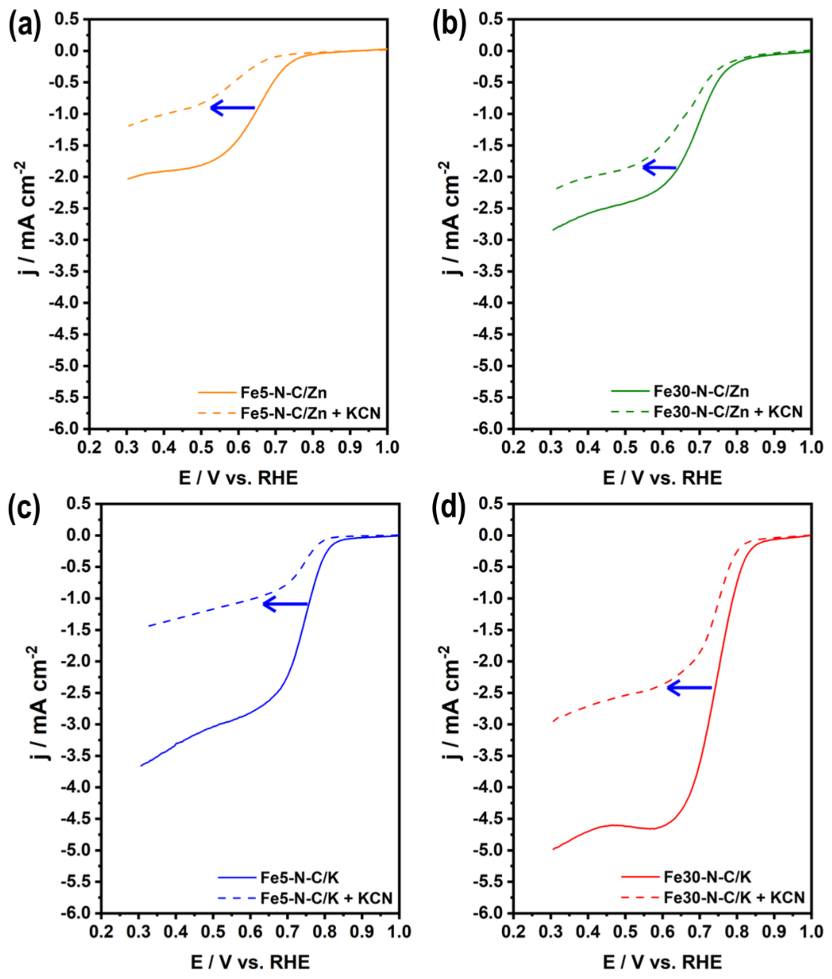


**Figure S6.** ORR polarization curves for **WP**/Fe-doped derived catalyst-modified GC electrodes in 0.1 M KOH solution saturated with $O_2$ (solid lines) and with the addition of 10 mM KCN (dashed lines) (scan rate: 5 $mVs^{-1}$, rotation rate: 1600 rpm).

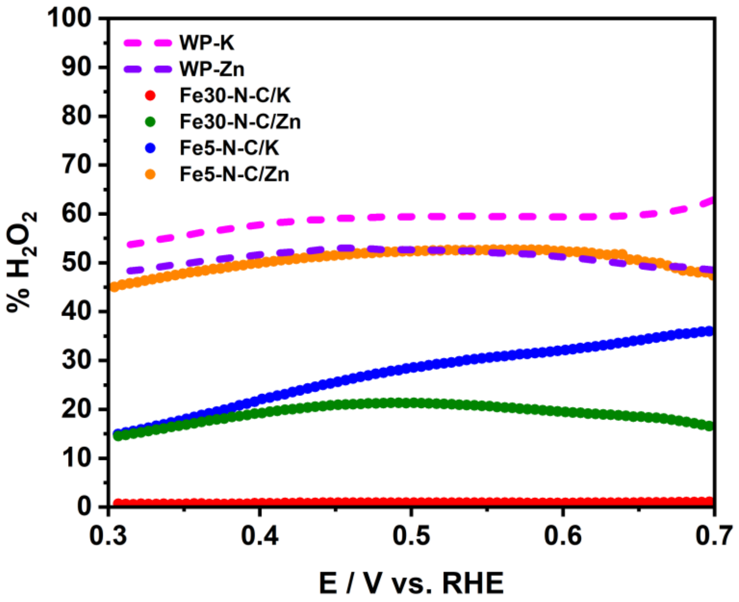


**Figure S7.** Hydrogen peroxide percentage yields as a function of potential detected by RRDE during the ORR on catalyst-coated GC electrodes in $O_2$-saturated 0.1 M KOH.
